# Characterization and Mechanism of Linearized-Microcystinase Involved in Bacterial Degradation of Microcystins

**DOI:** 10.3389/fmicb.2021.646084

**Published:** 2021-03-30

**Authors:** Jia Wei, Feiyu Huang, Hai Feng, Isaac Yaw Massey, Tezi Clara, Dingxin Long, Yi Cao, Jiayou Luo, Fei Yang

**Affiliations:** ^1^Hunan Provincial Key Laboratory of Clinical Epidemiology, Xiangya School of Public Health, Central South University, Changsha, China; ^2^Hunan Province Key Laboratory of Typical Environmental Pollution and Health Hazards, School of Public Health, University of South China, Hengyang, China; ^3^Key Laboratory of Environmental Medicine Engineering, School of Public Health Southeast University, Ministry of Education, Nanjing, China

**Keywords:** linearized-microcystinase, microcystins, biodegradation, homology modeling, molecular docking

## Abstract

Microcystins (MCs) are extremely hazardous to the ecological environment and public health. How to control and remove MCs is an unsolved problem all over the world. Some microbes and their enzymes are thought to be effective in degrading MCs. Microcystinase can linearize microcystin-leucine-arginine (MC-LR) *via* a specific locus. However, linearized MC-LR is also very toxic and needs to be removed. How linearized MC-LR was metabolized by linearized-microcystinase, especially how linearized-microcystinase binds to linearized MC-LR, has not been defined. A combination of *in vitro* experiments and computer simulation was applied to explore the characterization and molecular mechanisms for linearized MC-LR degraded by linearized-microcystinase. The purified linearized-microcystinase was obtained by recombinant *Escherichia coli* overexpressing. The concentration of linearized MC-LR was detected by high-performance liquid chromatography, and linearized MC-LR degradation products were analyzed by the mass spectrometer. Homology modeling was used to predict the structure of the linearized-microcystinase. Molecular docking techniques on the computer were used to simulate the binding sites of linearized-microcystinase and linearized MC-LR. The purified linearized-microcystinase was obtained successfully. The linearized-microcystinase degraded linearized MC-LR to tetrapeptide efficiently. The second structure of linearized-microcystinase consisted of many alpha-helices, beta-strands, and colis. Linearized-microcystinase interacted the linearized MC-LR with hydrogen bond, hydrophobic interaction, electrostatic forces, and the Van der Waals force. This study firstly reveals the characterization and specific enzymatic mechanism of linearized-microcystinase for catalyzing linearized MC-LR. These findings encourage the application of MC-degrading engineering bacteria and build a great technique for MC-LR biodegradation in environmental engineering.

## Introduction

Microcystins (MCs) are cyclic heptapeptide hepatotoxins produced by a harmful algal bloom, including *Microcystis*, *Anabaena*, and *Planktothrix* (Zurawell et al., 2005; Li et al., 2011). There are over 270 identified isomer types of microcystins (Carmichael et al., 2018; Massey et al., 2020), and the most widely distributed and the most toxic is microcystin-leucine-arginine (MC-LR) (Gupta et al., 2003; Huisman et al., 2018). MC-LR has a stable cyclic structure, which includes MeAsp, Adda, and Mdha (Krishnamurthy et al., 1989). MC-LR is capable of presenting potential hepatic toxicity and tumor-promoting activity by inhibiting protein phosphatases 1 and 2A and altering the expression levels of microRNAs (Le Manach et al., 2018; Yang S. et al., 2018; Chen et al., 2019). The International Agency for Research on Cancer categorized MC-LR as a carcinogen (group 2B) (IARC, 2010). A guideline value of 1 μg/L MC-LR in drinking water was recommended by the World Health Organization (Who, 1998). Consequently, it is important to identify potential strategies to mitigate the toxicity and hazards of MCs effectively.

Conventional water treatments have limitations in removing MCs because of the stable and resistant cyclic structure (Tsuji et al., 2006). While the chemical treatments were usually not environmentally friendly owing to their adverse effects on aquatic ecosystems (Yu et al., 2009; Li et al., 2017), the physical treatments were usually too costly to apply (Yu et al., 2009; Duan et al., 2018). Biological treatments against MC-LR were cost-effective ways of removing MC-LR from water systems (Dziga et al., 2016; Dimpe et al., 2017; Wei et al., 2020). A few single MC-degrading bacteria have been obtained, such as *Sphingopyxis* sp. USTB-05 (Yan et al., 2012), IM-1 (Lezcano et al., 2016), X20 (Qin et al., 2019), and YF1 (Yang et al., 2020), *Novosphingobium* sp. THN1 (Jiang et al., 2011), MD-1 (Okano et al., 2020), *Sphingomonas* sp. ACM-3962 (Bourne et al., 1996), Y2 (Park et al., 2001), and NV-3 (Ishii et al., 2004), *Bordetella* sp. MC-LTH1 (Yang et al., 2014). However, there are potential risks that these microorganisms used in treating MCs may become ecologically dominant or secrete unknown toxic substances into the water (Liu et al., 2020). Therefore, safe and effective treatment against MCs is still a great challenge.

*Sphingomonas* sp., N*ovosphingobium* sp., and *Sphingopyxis* sp. have been testified to contain MC-biodegrading gene cluster *mlrABCD*, which encoded MlrABCD enzymes (Bourne et al., 1996; Park et al., 2001; Ishii et al., 2004; Yan et al., 2012; Yang et al., 2020; Dexter et al., 2021). These enzymes play an important role in biodegrading MCs (Bourne et al., 1996, 2001, 2006; Saito et al., 2003; Dexter et al., 2021). It has been demonstrated that quorum sensing systems positively regulated the degradation of MC by the transcriptional induction of these MC-degrading genes, especially *mlrA* (Zeng et al., 2020). It is well testified that the enzyme of MlrA (also known as microcystinase) hydrolyzes the peptide bond between Adda and Arg of cyclic MCs, which significantly reduced the toxicity of MCs by linearized MCs (Bourne et al., 1996). However, linearized MC-LR still is very toxic; the 50% inhibitory concentration for protein phosphatase was 95 μM (Bourne et al., 1996). Therefore, it is necessary to reduce the concentration and distribution of linearized MC-LR. MlrB (also known as linearized-microcystinase) and MlrC (also known as tetrapeptidease) are in charge of biodegrading the linearized MCs (Dziga et al., 2012a, 2016; Shimizu et al., 2012; Xu et al., 2015; Yang et al., 2020). MlrD usually facilitates the moving of MCs, and its biodegradation products pass through the bacteria (Bourne et al., 2001). Hydrolysis of the peptide bond, demethylation, decarboxylation, and dehydration reactions participate in biodegrading MCs (Valeria et al., 2006; Edwards et al., 2008; Hashimoto et al., 2009; Dziga et al., 2017). However, the characteristics of biodegradation enzymes have not yet been described, and the biodegradation mechanisms are needed to be explored further.

The knowledge of the molecular process of MC metabolized by the bacteria is limited. MC-degrading bacteria are always carrying *mlr* gene cluster. Bourne et al. (1996) and Dziga et al. (2012b) found the enzyme microcystinase from MC-degrading bacterial *Sphingomonas* sp. ACM-3962 was probably a metalloprotease, and the metalloprotease contains an H^260^AIH^263^NE^265^ active center. In addition, a variant of the zinc-binding motif (HEXXH) was found in this metalloprotease typically. Xu et al. (2019) showed that microcystinase is likely not a metalloprotease but glutamate protease belonging to type II CAAX prenyl endopeptidases. The study on linearized-microcystinase speculated that the serine protease was strong, similar to members of the penicillin-recognizing enzyme, after comparing the sequence (Bourne et al., 2001). However, the structural basis for linearized-microcystinase and linearized-microcystinase attacking the peptide bond of linearized MC-LR are still unclear. This work aimed to heterologously express *linearized-microcystinase* derived from the indigenous bacteria *Sphingopyxis* sp. YF1 and investigate the characterization and enzymatic mechanism of linearized MC-LR biodegradation by linearized-microcystinase. The purified linearized-microcystinase was obtained by recombinant *Escherichia coli* overexpressing. The biodegradation effect and products of linearized-microcystinase against linearized MC-LR were investigated by high-performance liquid chromatography (HPLC) and mass spectrometery (MS). Homology modeling was applied to predict the structure of linearized-microcystinase. Linearized MC-LR as a ligand was used to build binding modes with linearized-microcystinase by molecular docking techniques and then explore the potential binding interactions. This work specifies the role of linearized-microcystinase in the bacterial utilization of MC-LR and provides a powerful evidence in the mechanism of enzymes that act on MC biodegradation.

## Materials and Methods

### Reagents

An MC-degrading bacterial strain of *Sphingopyxis* sp. YF1 isolated from Lake Taihu was cultured in nutrient broth medium [3-g beef extract, 5-g peptone, and 5-g sodium chloride (NaCl) per 1,000 ml at pH 7.0]. *E. coli* BL21 (DE3) and *E*. *coli* DH5α were cultured in Luria–Bertani (LB) medium (10-g tryptone, 5-g yeast extract, and 10-g NaCl per 1,000 ml at pH 7.2).

Standard MC-LR with purity ≥95% was purchased from Taiwan Algal Science Inc. (Taiwan, China) and stored at −20°C. Trifluoroacetic acids and methanol were purchased from Dikma Technology Inc. (CA, United States) and used for HPLC analysis. The prokaryotic expression vector pGEX-4T-1, *E*. *coli* BL21 (DE3), and *E*. *coli* DH5α were purchased from Vazyme (Nanjing, China). T4 DNA ligase, Phusion High-Fidelity DNA Polymerase, BamH I, and Xho I were obtained from Thermo Fisher Scientific (MA, United States).

### Verification of Coding Sequence of *Linearized-Microcystinase*

The protein encoded by the *linearized-microcystinase* gene of *Sphingopyxis* sp. YF1 was termed linearized-microcystinase (Yang et al., 2020). The *Sphingopyxis* sp. YF1’s genomic DNA was extracted based on the genomic DNA extraction kit (Tiangen, Beijing, China). Two specific oligonucleotide primers (*linearized-microcystinase*-*BamH*I-F: 5′- CGC-GGATCCATGCCAATGGC GACTT-CCC-3′, *linearized-microcystinase*-*Xho*I-R: 5′- CCG-CTCGAGCTAC-GG-AAGCCGTCTG-3′) designed by Primer-Blast (Ye et al., 2012) were synthesized from Sangon Biotech (Shanghai, China). The specific primers were used for polymerase chain reaction (PCR) to detect the *linearized-microcystinase* gene from *Sphingopyxis* sp. YF1. The underlined sequences are *BamH* I and *Xho* I restriction sites, respectively. PCR was performed in a total volume of 50 μl with the following conditions: 25 μl 2 × Phanta Max Buffer, 1 μl deoxynucleoside triphosphate, 2 μl forward primer, 2 μl reverse primer, 1 μl DNA template, 1 μl Phanta Max Super-Fidelity DNA Polymerase, and 18 μl double-distilled water. The PCR runs with an initial denaturation at 95°C for 3 min, 35 thermal cycles of denaturation for 15 s at 98°C, annealing for 15 s at 56°C, an extension for 1 min at 72°C, and a final extension for 5 min at 72°C. Sequence similarity searches were conducted using the National Center for Biotechnology Information (NCBI) BLAST network service^1^.

### Phylogenetic Analysis of *Linearized-Microcystinase*

Multiple gene sequences of *linearized-microcystinase* were obtained from published research. The tree based on ClustalW alignments of the nucleotide sequences was built with the neighbor-joining method on MEGA7 software (Kumar et al., 2016). The numbers at the nodes are bootstrap support levels (%) based on the neighbor-joining analyses of 1,000 resampled datasets.

### Construction of Recombinant Plasmids pGEX-4T-1-*Linearized-Microcystinase*

Plasmids were extracted from *E*. *coli* DH5α and *E*. *coli* BL21 (DE3) using the small plasmid extraction kit (Tengen, Beijing, China). The PCR product and pGEX-4T-1 vector were sheared by *BamH* I and *Xho* I for 3 h at 3°C and ligation with T4 DNA ligase for 24 h. Then, the licensed products were transferred into *E*. *coli* DH5α. The recombinant plasmids were sequenced and identified using the NCBI BLAST network service.

### Expression and Purification of Recombinant pGEX-4T-1-*Linearized-Microcystinase*

*E. coli* BL21 (DE3) cells were transferred with pGEX-4T-1-*linearized-microcystinase* and plated on LB agar containing 100 μg/ml ampicillin. A single colony was cultured into liquid LB medium with 100 μg/ml ampicillin and shaken at a constant condition of 37°C, 200 rpm. Isopropyl-β-D-thiogalactopyranoside (IPTG), with the final concentration of 0.2 mM, was added to induce protein expression when the optical density at 600 nm was approximately 0.6. Also, the bacterium was constantly incubated at 30°C, 200 rpm for 3 h. The *E*. *coli* cells were harvested by centrifuging (8,000 × *g*, 20 min, 4°C), and the cells were resuspended in phosphate-buffered saline (PBS, pH = 7.0) three times. Cells were placed on ice and disrupted by sonication, and the resultant was centrifuged (15,000 × *g*, 20 min, 4°C). The supernatant was purified by applying to the GST-Accept nickel column (TaKaRa, Otsu, Japan). The columns were washed twice with the washing buffer at pH 8.0, which contained 50 mM Tris hydrochloride (Tris-HCl) and 150 mM NaCl, and then, the bound proteins were eluted from the columns with an elution buffer (50 mM Tris-HCl, 150 mM NaCl, 250 mM glutathione, pH 8.0). The protein profile was analyzed using sodium dodecyl sulfate-polyacrylamide gel electrophoresis (SDS-PAGE) on 10% polyacrylamide gel. Also, the concentration of the purified protein was determined at 280 nm using the Ultramicro spectrophotometer (IMPLEN P330, Munich, Germany).

### Enzymatic Activity Detection of Recombinant pGEX-4T-1-*Linearized-Microcystinase*

Linearized MC-LR was obtained by incubating MC-LR with 0.76 mg/ml microcystinase from strain YF1 for 20 min (Yang et al., 2020). To detect the activity of recombinant protein linearized-microcystinase, the protein was incubated in 10-mM PBS at 30°C and pH 7. Then, 0.76 mg/ml linearized-microcystinase was added to 10-mM PBS solution containing 2.5, 5.0, 10.0, 20.0, and 40.0 μg/ml linearized MC-LR and incubated at 30°C, 200 rpm (Yan et al., 2012; Yang et al., 2020). Controls were prepared in the same way but without linearized-microcystinase. Fifty-microliter samples were taken at 20-min intervals and centrifuged (12,000 × *g*, 15 min, 4°C) for monitoring the linearized MC-LR concentrations by HPLC. All experiments were performed in triplicate. The kinetic parameters of linearized-microcystinase reaction were analyzed by GraphPad Prism (GraphPad Software Inc., San Diego, CA).

### Analysis of Linearized Microcystin-Leucine-Arginine and Its Degrading Products

The concentration of linearized MC-LR and its degradation products were measured using the Agilent 1100 HPLC machine (Agilent, Palo Alto, CA, United States) with a Zorbax Extend C18 column (4.6 × 150 mm, 5 μm) and a variable wavelength detector set at 238 nm. The mobile phase was a mixture of 0.05% trifluoroacetic acid aqueous solution and methanol (37:63, v/v) at a flow rate of 0.8 ml/min. The injection volume was 10 μl, and the column temperature was maintained at 40°C.

Linearized MC-LR and degradation products were identified by HPLC, coupled with an ultra-high-resolution LTQ Orbitrap Velos Pro ETD MS (Thermo Scientific, Germany) and equipped with electrospray ionization interface (HPLC-ESI-MS) (LTQ Orbitrap Velos Pro ETD, Thermo Fisher, United States). The analysis condition of HPLC-ESI-MS was described previously (Yang et al., 2020).

### Homology Modeling and Molecular Docking

Characteristics of linearized-microcystinase were predicted by the “normal” mode of the Phyre2 engine (Kelley et al., 2015) when inputting the full-length amino acid sequences of linearized-microcystinase. Another professional web server for protein homology modeling, including I-TASER (Yang and Zhang, 2015), was examined for comparison as well. Both Phyre2 and I-TASER engines modeled the linearized-microcystinase structures based on the same template. Only the results of I-TASER were shown in the present report. The molecular structure of the linearized-microcystinase docking ligand linearized MC-LR was drawn by ChemOffice software. Blind docking of linearized MC-LR to linearized-microcystinase was counted by Autodock Vina (Morris et al., 2009). Pymol 2.3 (DeLano, 2002) was used to visualize the three-dimensional (3D) structure of the optimal binding model, and Discovery Studio v2.5 (Accelrys Software Inc., San Diego, CA, United States) was used to analyze the interaction pattern and active sites between the receptor and the ligand.

## Results

### Sequencing and Phylogenetic Analysis of *Linearized-Microcystinase*

**FIGURE 1:**
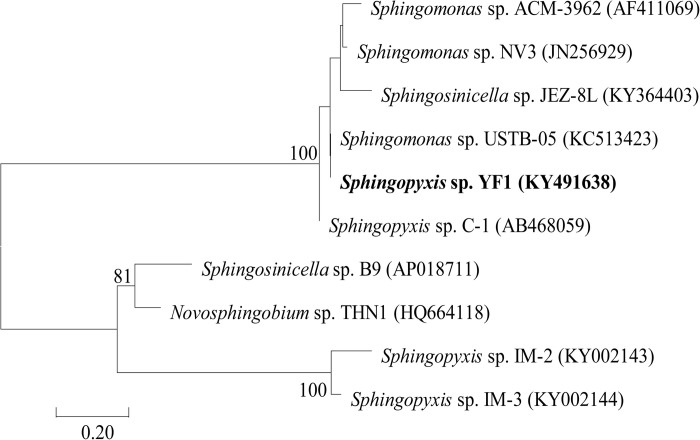
A phylogenetic tree based on the *linearized-microcystinase* sequence.

When the *linearized-microcystinase*-specific primers were used for PCR, the size of products was 1,584 bp. Nucleotide-nucleotide BLAST analysis (blastn) on the web of NCBI was conducted by placing a *linearized-microcystinase* sequence. According to blastn analysis and the phylogenetic tree based upon its *linearized-microcystinase* sequence, the *linearized-microcystinase* (KY491638) nucleotide sequence from the strain YF1 was 99.35% similar to *linearized-microcystinase* from *Sphingomonas* sp. USTB-05 (KC513423) (Figure 1).

### Expression and Purification of Linearized-Microcystinase

**FIGURE 2:**
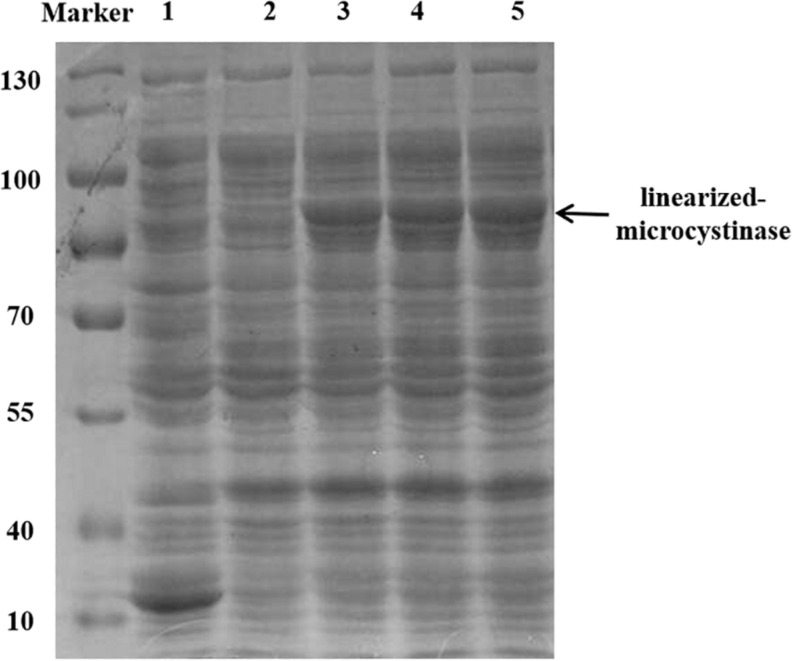
Purification and SDS-PAGE of the linearized-microcystinase; 1: pGEX-4T-1; 2: uninduced linearized-microcystinase. 3–5: induce linearized-microcystinase

The *linearized-microcystinase* gene from the indigenous bacteria *Sphingopyxis* sp. YF1 was introduced into a pGEX-4T-1 vector and overexpressed in the *E. coli* DH5α strain. The purified linearized-microcystinase protein was successfully obtained, exhibiting greater than 90% purity. Using SDS-PAGE analysis, a clear overexpressed band amount to a molecular approximately weight of 85 kDa after IPTG induction was observed (Figure 2).

### Enzymatic Activity and Degradation Products of Linearized Microcystin-Leucine-Arginine

After the purified protein linearized-microcystinase was incubated with linearized MC-LR, the linearized MC-LR was efficiently degraded. When the concentrations of linearized MC-LR were 2.5, 5.0, 10.0, 20.0, and 40.0 μg/ml, the maximum linearized MC-LR degradation velocities were up to 1.2, 1.8, 2.4, 3.5, and 4.6 μg/(L⋅h), respectively (Supplementary Figure 1). The degradation velocity increased with the linearized MC-LR concentrations (Supplementary Figure 1). The kinetic constants Michaelis constant (Km) and maximal velocity (Vmax) of the linearized-microcystinase obtained by Michaelis–Menten Equation were 10.03 ± 1.88 μM and 5.6 μg/(L⋅h), respectively.

**FIGURE 3:**
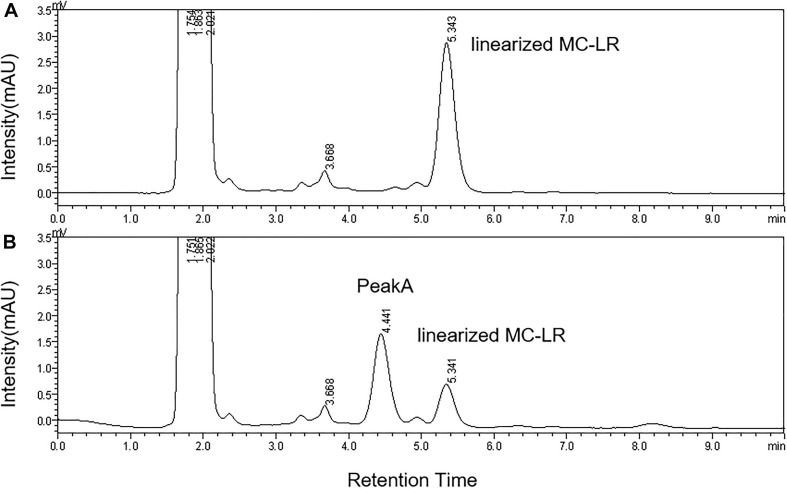
HPLC analysis of the degradation of linearized MC-LR incubated with the linearized-microcystinase at time 0 min **(A)** and 20 min **(B)**.

**FIGURE 4:**
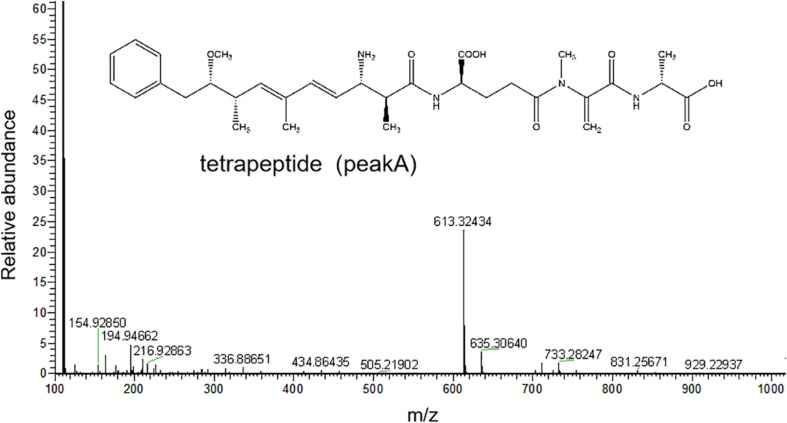
HPLC-ESI-MS spectrum in the negative mode and the molecular structure of the product A of linearized MC-LR.

The HPLC chromatography showed that the retention time of the linearized MC-LR was 5.3 min. The peak area of linearized MC-LR declined significantly after incubation with linearized-microcystinase. The main intermediate degradation products of linearized MC-LR (peak A) had a retention time of 4.4 min (Figure 3). The linearized MC-LR and its degradation products were further determined using the HPLC-ESI-MS system. MS analysis in the negative mode revealed the molecular ion of peak A at m/z 613.32434[M + H] +, which was in agreement with the calculated mass of C_32_H_46_N_4_O_8_, identified as tetrapeptide (Figure 4).

### Homology Modeling of Linearized-Microcystinase

**FIGURE 5:**
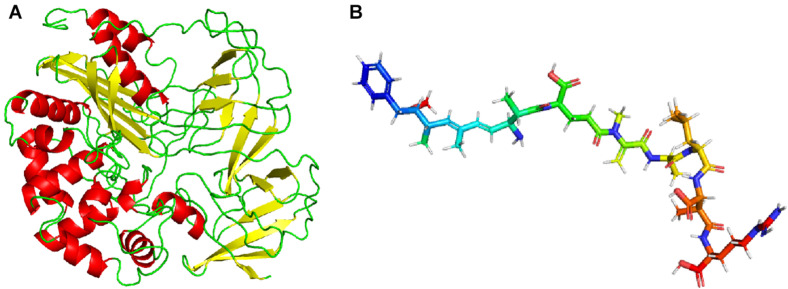
Homology modeling of linearized-microcystinase **(A)** and linearized MC-LR **(B)**.

I-TASER and PHYRE 2 web servers were used to predict the structure of linearized-microcystinase, and the multiple domains of this protease were the models. Both I-TASER and PHYRE 2 servers have high confidence and scoring based on template 1ei5A. The model was successfully constructed with 0.39 of C-score, 0.74 ± 0.11 TM score, and 7.0 ± 4.1 Å root-mean-square deviation score (Figure 5A). Homology modeling results showed that the secondary structure of linearized-microcystinase consisted of 12 alpha-helices, 18 beta-strands, and 26 colis (Figure 5A). The 3D structure of the linearized-microcystinase predicted by the I-TASER server presented a 99.6% confidence level. The N-terminal and C-terminal regions could be located on the cell surface with one transmembrane domain S1 linked by a reentrant helix, whereas the transmembrane helices were predicted to adopt the topology (Supplementary Figure 2). The model of ligand linearized MC-LR was drawn using ChemOffice (Supplementary Figure 3) and, after the energy optimization, the final ball-and-stick model as shown in Figure 5B.

### Molecular Docking and Active Sites Analysis

**FIGURE 6:**
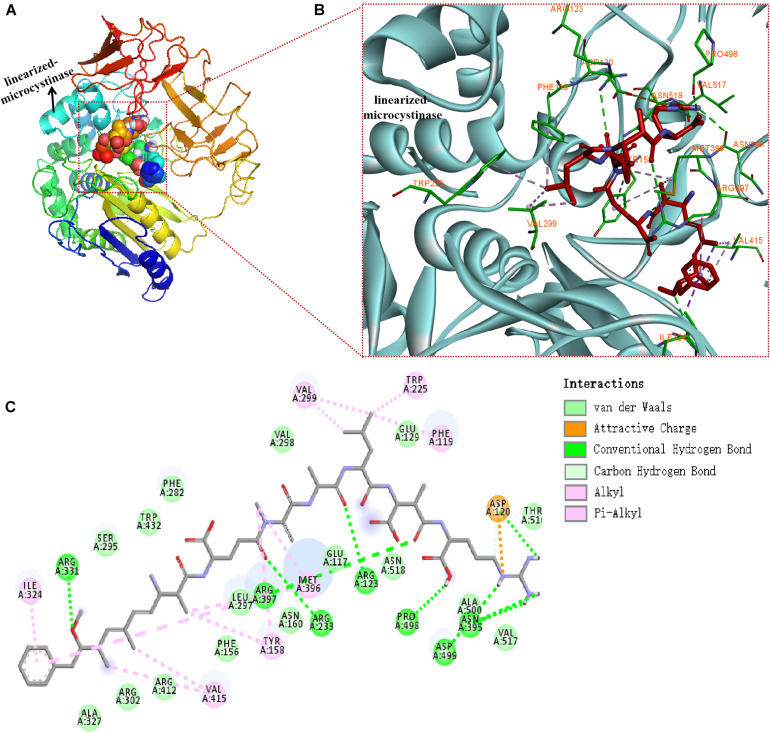
Docking model of linearized-microcystinase and linearized MC-LR. **(A)** Overall model of linearized-microcystinase and linearized MC-LR. **(B)** 3D view of the predicted interactions between linearized-microcystinase and linearized MC-LR. Linearized MC-LR is shown in red, the linearized-microcystinase protein is shown in cyan, whereas the amino acids potentially involved in linearized-microcystinase and linearized MC-LR interaction are shown in green (with orange label). Green, pink, and orange dotted lines indicated the formation of hydrogen bonds, hydrophobic, and electrostatic, respectively. **(C)** 2D view of the predicted interactions between linearized-microcystinase and linearized MC-LR.

The binding mode of linearized-microcystinase and linearized MC-LR was revealed by molecular docking (Figure 6). The model of linearized-microcystinase that consisted of conserved amino acid residues formed a pocket, which provided a possibility of an active site for the protein (Figure 6A). After analyzing the active site by binding site of AutoDock Vina, we got 20 possible active center sites, and the lowest energy conformation was selected (Figure 6A). The results indicated that linearized MC-LR was successfully docked into the binding pockets of linearized-microcystinase with a docking score of -8.5 kcal/mol. The pose with the lowest score, suggesting the most probable binding modes of a ligand, was selected for further analysis (Figure 6). The binding poses of the linearized MC-LR and linearized-microcystinase were assessed using the scoring function that comprised Van der Waals energy, coulomb energy, hydrophobic interactions, hydrogen bonding, polar interactions, and rotatable bonds penalty. The detailed protein-ligand interactions of the docking pose (3D and 2D structures) suggested the involvement of 8 hydrogen bonds, 11 hydrophobic and 1 electrostatic (Figures 6B,C). These interactions include (i) conventional hydrogen bonding with ARG^331^, ARG^397^, ARG^233^, ARG^123^, PRO^498^, ASP^499^, ASP^120^, and ASN^395^; (ii) Pi-Alkyl bond with ILE^324^, VAL^299^, TRP^225^, PHE^119^, MET^396^, TYR^158^, and VAL^415^; and (iii) attractive charge interactions with ASP^120^. In addition, linearized-microcystinase strongly interacted with linearized MC-LR through Van der Waals force, which involved 16 amino acid residues (Figure 6C).

## Discussion

Biodegradation is important for the reduction of MCs in eutrophic water. Both microbial community and single strain were demonstrated to have the ability to degrade MCs (Bourne et al., 1996; Ishii et al., 2004; Yan et al., 2012; Lezcano et al., 2016; Yang F. et al., 2018; Yang et al., 2020). However, this work mostly emphasized the effects of bacteria that degrade MCs. Microorganisms used in biological treatments may also carry potential risks (e.g., biological invasion) (Liu et al., 2020). Less information is available on the effects of a secreted substance (e.g., active enzyme) on the degradation of MCs. It is the first time to investigate the characterization and molecular mechanism of linearized-microcystinase that degrade linearized MC-LR.

The phylogenetic tree analysis that showed the *linearized-microcystinase* gene from a novel bacterial *Sphingopyxis* sp. YF1 was analogical to *linearized-microcystinase* of MC-degrading bacteria *Sphingomonas* sp. USTB-05. Therefore, the linearized-microcystinase encoded by the *linearized-microcystinase* gene from bacterium YF1 may participate in degrading MC-LR (Bourne et al., 2001). Purified enzymes were often used to study their functions and characteristics (Zhang et al., 2020). Heterologously expressed MC-degrading proteins were very useful in investigating the biochemical pathways and subsequent MC derivatives. Recombinant MC-degrading proteins have been applied in this study. The purified linearized-microcystinase was successfully obtained from the strain YF1 by recombinant *E. coli* overexpressing. Here, the most important gene (*linearized-microcystinase*, 1,584 bp) of *Sphingopyxis* sp. YF1 that involved in the biodegradation of linearized MC-LR was firstly cloned and successfully expressed. Enzymatic degradation of MCs, however, caught our attention, as indicated in this investigation. After incubation with linearized MC-LR, linearized-microcystinase degraded the substrate efficiently, and the degradation rate increased as linearized MC-LR concentration rose (Supplementary Figure 1). Michaelis constants of the enzyme linearized-microcystinase were = 10.03 ± 1.88 μM. According to the previous report, the Michaelis constant is a vital index, which plays an important role in determining the specificity, efficiency, and proficiency of enzymes (Johnson, 2013). Therefore, the Michaelis constants were helpful indicators for people to compare the efficiency of different MC-degrading enzyme homologs in the future (Yang et al., 2020). The maximum biodegradation velocities (Vmax) obtained from the calculation was 5.617 μg/ml. Dziga et al. (2012a) found that recombinant linearized-microcystinase from *Sphingomonas* sp. ACM-3692 could hydrolyze linearized MC-LR, but the significant linearized MC-LR degradation rate by linearized-microcystinase has not been observed. In the process of degradation, the concentration of linearized MC-LR decreased, and an intermediate product appeared, which indicated that the linearized-microcystinase degraded linearized MC-LR into a smaller molecule. The intermediate product (peak A) was collected; then, it was identified as tetrapeptide (C_32_H_46_N_4_O_8_) by MS. These results were consistent with the previous study, Bourne et al. (1996), Dziga et al. (2016), and Yang et al. (2020), which also reported that linearized MC-LR was degraded to tetrapeptide by some MC-degraded bacteria according to HPLC-MS data. Bourne et al. (2001), through cloning and screening gene library of *Sphingomonas* sp. strain, found that linearized MC-LR could be degraded by the enzymes of linearized-microcystinase and tetrapeptidease. However, the degradation rate of linearized MC-LR by linearized-microcystinase and the specific binding sites between linearized-microcystinase and linearized MC-LR has not been explored.

Although some reports showed that the degradation of MC associated with the *mlr* gene cluster, the function of linearized-microcystinase remains unclear (Bourne et al., 1996, 2001; Harada et al., 2004; Imanishi et al., 2005; Dziga et al., 2016; Zeng et al., 2020). How linearized MC-LR was metabolized by linearized-microcystinase, especially how linearized-microcystinase binds to linearized MC-LR, has not been defined. In this study, the linearized-microcystinase has an active pocket that is particularly suited for the presence of linearized MC-LR. Linearized MC-LR was degraded by linearized-microcystinase *via* strongly multiple hydrogen bonding, hydrophobic and electrostatic interactions, and Van der Waals forces. It was the first time to explore the interaction and specific binding sites between linearized-microcystinase and linearized MC-LR. Many amino acid residues of linearized-microcystinase, including ARG ^331^, ASP ^499^, ILE^324^, VAL^299^, PHE^119^, TYR^158^, VAL^415^, and so on, were involved in forcing linearized MC-LR into tetrapeptide. Molecular docking has emerged as a powerful tool to explore the molecular mechanisms of the protein–ligand, protein–nucleotide, and protein–protein interactions, and this technique is often applied to predicted possible binding pattern of the substrates against its bioactive molecule (Pingaew et al., 2018; Zulkipli et al., 2020). Before simulation of molecular docking with computers, we need to get the structure of linearized-microcystinase. The homology modeling was used to predict the structure of linearized-microcystinase based on similar amino acid sequences because the crystal structure of linearized-microcystinase has not been determined yet. C-score in the range of -5 and 2 is a confidence score for estimating the high quality of predicted models by I-TASSER (Yang and Zhang, 2015; Yang et al., 2015). The model of linearized-microcystinase base on a template 1ei5A in this study has high confidence with 0.39 C-score. It was possible to use this template for further analysis. After docking, the calculated docking score for the linearized-microcystinase model and linearized MC-LR was -8.5 kcal/mol, which indicated a good binding affinity between them (Zulkipli et al., 2020). Generally, the interaction between the ligands and receptors probably included hydrogen bonds, Van der Waals forces, electrostatic forces, hydrophobic interactions, steric contact, and so on (Leckband, 2000; Hou et al., 2019). They are regarded as the main forces acting as small ligands binding to receptors. Moreover, the combination of 2D and 3D models help to predict the biological activities of the proteins more accurately (Tran et al., 2020).

The linearized-microcystinase from strain YF1 probably has the same function as other MC-degrading bacteria because their amino acid sequences were similar. Recombinant *E. coli* overexpressing demonstrated that the linearized-microcystinase was an important enzyme involved in MC degradation. The 50% protein phosphatase inhibition rate of linearized MC-LR was 95 μM (Bourne et al., 1996). Hence, linearized MC-LR is very toxic to animals and humans. Linearized-microcystinase can promote the degradation of MC-LR, which played an important role in detoxifying MC-LR (Bourne et al., 2001; Dziga et al., 2016; Yang et al., 2020). Tetrapeptide is also toxic, but it is an essential intermediate in the Linearized MC-LR detoxification process. Linearized MC-LR was firstly degraded into tetrapeptide, then turned into Adda, phenylacetic acid, and finally potential non-toxic product CO_2_. In addition, the exact active binding site of linearized-microcystinase to degrade linearized MCs needs to be testified. For example, knockout gene experiments and site-directed mutation experiments are required to better understand the degradation pathway. How to make MC-degrading-related enzymes available for application in polluted water needs further study. Here, we found a new way to remove linearized MC-LR efficiently using the single enzyme linearized-microcystinase. In the future, modify linearized-microcystinase enzymes for convenient preservation so that they can be used in MC polluted water. It is also a great way to complete the degradation of MCs by constructing engineered bacteria and fixing microcystinase, linearized-microcystinase, and tetrapeptidease into some special materials.

## Conclusion

In this study, linearized-microcystinase, an enzyme coded by *linearized-microcystinase*, with linearized MC-LR degrading ability, was successfully obtained through overexpression in *E. coli*. With the aim of describing the characterization and molecular mechanism of linearized-microcystinase degrading linearized MC-LR, we first investigated the interaction of linearized-microcystinase and linearized MC-LR by homology modeling and molecular docking technology. Linearized-microcystinase probably consisted of alpha-helices, beta-strands, and colis, and it efficiently altered linearized MC-LR to tetrapeptide. The dominant binding forces of linearized-microcystinase and linearized MC-LR were hydrogen bond, hydrophobic interaction, electrostatic forces, and Van der Waals force. This study provides a better understanding of the enzymatic mechanism of linearized MC biodegradation by linearized-microcystinase. Linearized-microcystinase may be useful in the complete elimination of the hepatotoxic toxins in the water.

## Data Availability Statement

The datasets presented in this study can be found in online repositories. The names of the repository/repositories and accession number(s) can be found in the article/Supplementary Material.

## Author Contributions

JW, JL, FH, and FY conceived and designed the experiments. JW and FH performed the experiments. JW, HF, and FY analyzed the data. FY and JL contributed reagents, materials, and analysis tools. JW, IM, TC, JL, and FY wrote and revised the manuscript. All authors contributed to the article and approved the submitted version.

## Conflict of Interest

The authors declare that the research was conducted in the absence of any commercial or financial relationships that could be construed as a potential conflict of interest.

## Footnotes

1 http://www.ncbi.nlm.nih.gov/BLAST

## Abbreviations

ARG: arginine
ASN: asparagine
ASP: aspartic acid
ILE: isoleucine
MET: methionine
PHE: phenylalanine
PRO: proline
TRP: tryptophan
TYR: tyrosine
VAL: valine.
