# Supplementary material for: Characterization and Mechanism of Linearized-Microcystinase Involved in Bacterial Degradation of Microcystins
Source: Front Microbiol. 2021 Mar 30;12:646084. doi: 10.3389/fmicb.2021.646084 (PMC8042282; doi:10.3389/fmicb.2021.646084)
Supplement: Supplementary file 1 [file Data_Sheet_1.docx]

**Characterization and Mechanism of Linearized-microcystinase Involved in Bacterial Degradation of Microcystins**

Jia Wei 1, Feiyu Huang ^1^, Hai Feng ^1^, Isaac Yaw Massey ^1^, Tezi Clara ^1^, Dingxin Long ^2^, Yi Cao ^2^, Jiayou Luo ^1^, Fei Yang ^1, 2, 3*^

^1^ Hunan Provincial Key Laboratory of Clinical Epidemiology, Xiangya School of Public Health, Central South University, Changsha 410078, China;

^2^ Hunan Province Key Laboratory of Typical Environmental Pollution and Health Hazards, School of Public Health, University of South China, Hengyang 421001, China;

^3^ Key Laboratory of Environmental Medicine Engineering, Ministry of Education, School of Public Health Southeast University, Nanjing 210009, China;

*Corresponding author. E-mail: yangfeilong@126.com (F.Y.)


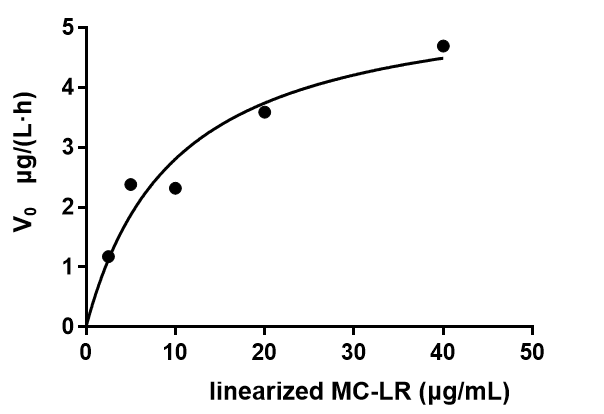


Figure S1. Effect of initial linearized MC-LR concentration on the degradation velocity of linearized MC-LR by linearized-microcystinase.


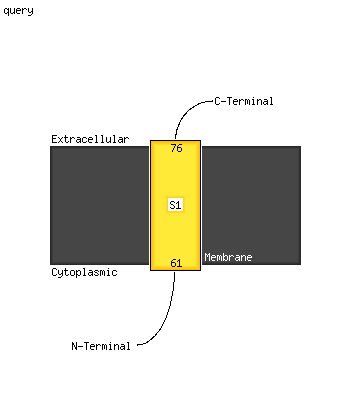


Figure S2. Predicted transmembrane helix regions of microcystinase by Phyre 2. Cell membrane is presented as the gray shaded area. S1 is transmembrane domains of linearized-microcystinase.





Figure S3. Chemical structure of linearized MC-LR.
